# Lead ferrite-activated carbon magnetic composite for efficient removal of phenol from aqueous solutions: synthesis, characterization, and adsorption studies

**DOI:** 10.1038/s41598-022-15077-x

**Published:** 2022-06-23

**Authors:** Esmaeil Allahkarami, Abolfazl Dehghan Monfared, Luis Felipe Oliveira Silva, Guilherme Luiz Dotto

**Affiliations:** 1 Department of Petroleum Engineering, Faculty of Petroleum, Gas and Petrochemical Engineering, Persian Gulf University, Bushehr, 75169-13817 Iran; 2 Department of Civil and Environmental, Universidad de La Costa, CUC, Calle 58 # 55-66, Barranquilla, Atlántico Colombia; 3 Chemical Engineering Department, Federal University of Santa Maria, UFSM, Roraima Avenue 1000, Santa Maria, RS 97105900 Brazil

**Keywords:** Chemical engineering, Engineering

## Abstract

A novel lead ferrite-magnetic activated carbon (lead ferrite-MAC) composite was developed using the chemical co-precipitation method. Instrumental analyses such as X-ray diffraction (XRD), scanning electron microscopy (SEM), Fourier-transform infrared spectroscopy (FTIR), and Brunauer–Emmett–Teller (BET) analysis were performed to characterize adsorbent. The uptake of phenol from aqueous solutions using the developed adsorbent was compared to that of pristine activated carbon. The maximum adsorption capacity of lead ferrite-MAC composite (145.708 mg/g) was more than that of pristine activated carbon (116.606 mg/g) due to the metal hydroxides coated on activated carbon since they improve the retention of phenol on the available active sites of adsorbent and create an additional electrostatic interaction with the phenol adsorbate. Regarding the high value of the coefficient of determination (*R*^2^) and adjusted determination coefficient (*R*^2^_*adj*_), coupled with the lower values of average relative error (*ARE*) and minimum squared error (*MSE*), it can be found that the isothermal data for the lead ferrite-MAC adsorbent were in agreement with the isotherm models of Redlich-Peterson and Langmuir. From the kinetic viewpoint, pseudo-second-order and linear driving force models explained the phenol adsorption data for both adsorbents. The reusability tests for lead ferrite-MAC composite revealed that after six cycles, 85% of the initial adsorption capacity was maintained. The developed adsorbent can be successfully applied to uptake phenol from aqueous solutions.

## Introduction

Phenols are the most common contaminants generated by petrochemical units, coal gasification sites, and oil refineries^1, 2^. They are mainly applied to produce phenolic resins, adhesives and epoxy resins, and polyamide for different uses^3^. However, phenolic compounds are extremely toxic^4^, lead to unpleasant odor and taste of drinking water, and endanger living organisms^5, 6^. Therefore, they should be removed from the aqueous solution. Different approaches are used for removing phenolic compounds from waste streams, including activated sludge^7^, bioremediation^8^, membrane filtration^9^, solvent extraction^10^, and adsorption^11^. Table 1 shows the advantages/disadvantages of mentioned techniques. Adsorption is the most efficient method for wastewater decontamination applications due to its efficiency, selectivity, low operating cost, and high adsorption capacity. Also, it does not produce toxic substances^12, 13^.

**Table 1:** Advantages/disadvantages of methods used for the removal of phenol.

Method	Advantages	Disadvantages	References
Activated sludge	Relatively low cost Straightforward process	Inefficient for treating high phenol concentration The complexity of adsorbent regeneration	^30^
Bioremediation	Straightforward process Good selectivity	Inefficient for treating high molecular weight polycyclic aromatic hydrocarbons	^8, 31^
Solvent extraction	Good selectivity and efficiency Good process kinetics	Need for high solvent concentrations Relatively high cost	^32, 33^
Membrane filtration	Small space requirements, Efficient process	Process complexity Relatively high cost Pre-conditioning	^9, 34^
Adsorption	Simple process Efficient process Selective process Relatively low cost	Slow process pH dependence Non-destructive operation	^35^

There are different adsorbents such as minerals^14^, biological materials^15^, polymer materials^16^, and activated carbon^17^ to uptake phenol from different aqueous solutions. Koduru et al.^18^ examined the uptake of bisphenol A (BPA) from water using goethite/activated carbon composite in a batch system. According to their report, the kinetic and equilibrium data were in agreement with the pseudo-second-order model and Freundlich isotherm, respectively. Park et al.^19^ powdered activated carbons impregnated with iron oxide nanoparticles and used it to uptake bisphenol A and natural organic matter from the aqueous solution. They found that the pH of the solution had no significant effect on BPA removal, but acidic conditions gave a slightly reduced sorption capacity, possibly because of weaker hydrogen bonding between iron oxide and BPA^20^. Lingamdinne et al.^21^ synthesized magnetic inverse spinel iron oxide nanoparticles using a biogenic methodology and used it for the removal of pollutants from the aqueous solutions. The batch adsorption studies concluded that the adsorption of Pb(II) and Cr(III) was the monolayer adsorptions on the homogenous surface of the developed adsorbent. In addition, the kinetic data were in agreement with the pseudo-second-order kinetic model.

Promisingly, the application of activated carbon for wastewater decontamination purposes is of great interest in recent years. In this way, the fabrication and improvement of the sorbent properties is the topic of research. Din et al.^22^ fabricated a type of activated carbon from coconut shell (MNAC) and studied its application for the uptake of phenol. The rate-limiting step of phenol adsorption onto MNAC was a chemical reaction because their kinetic data were consistent with the pseudo-second-order model. According to the Langmuir isotherm, the maximum adsorption capacity for this adsorbent was found to be 205.8 mg/g. Iron oxide/carbon nanocomposites are frequently used as effective adsorbent materials to uptake different pollutants from the aqueous solutions^23^. Ianoş et al.^24^ investigated the adsorption of the dyes Acid Blue 129, Methylene Blue, Rhodamine 6G, and Acid Orange 7 on iron oxide/carbon nanocomposites. However, the development of metal ferrite coated carbon composites has gained great interest as adsorbents to remove contaminants^25, 26^. In general, ferrite materials are of significant interest due to their high adsorption capacity, non-toxicity, and easy availability^25^. Regarding the favorable capacity of such adsorbents, it is important to develop an activated carbon-based composite and evaluate its possible application for wastewater treatment purposes. Spinel metal ferrites (MFe_2_O_4_, M = Zn, Mn, Co, Pb, Ba, Sr, and so on) have face-centered cubic structures and M^2+^ and Fe^3+^ cations fill the coordination places in tetrahedral and octahedral. The arrangement of spinel ferrites influences their structural, chemical, and magnetic properties. On the other hand, the use of lead (Pb), barium (Ba), and strontium (Sr) in the composition of ferrite materials could improve the magnetic properties of the material. Magnetically modified carbon materials have received great attention for wastewater treatment purposes. Many reports are available regarding the preparation of magnetic carbon material, and these materials were applied for the removal of different pollutions from the aqueous medium. These reported magnetically modified carbon materials have agreeable advantages of easy recovery and high adsorption efficiency. Yang et al.^27^ prepared magnetic Fe_3_O_4_/AC for the uptake of methylene blue from aqueous solutions. The application of MnFe_2_O_4_ and CuFe_2_O_4_ composites for the uptake of contaminants were reported by Zhang et al.^28^. Feng^29^ prepared NiFe_2_O_4_/AC composite for the uptake of methylene blue, rhodamine B, and malachite green. There is no systematic research on the synthesize and application of lead ferrite-activated carbon composite for the removal of pollutants from waste streams. Thus, the main aim of this research is to develop a new adsorbent namely lead ferrite-activated carbon composite for the uptake of phenol from waste streams.

In this research, a novel lead ferrite-activated carbon composite (lead ferrite-MAC) was prepared using chemical co-precipitation. The phenol uptake using the developed adsorbent was compared to that of pristine AC. The most influential factors like phenol concentration, contact time, and solution acidity on the performance of phenol adsorption onto both adsorbents were separately investigated. In addition, X-ray diffraction (XRD), scanning electron microscopy (SEM), Fourier-transform infrared spectroscopy (FTIR), Brunauer–Emmett–Teller (BET) analyses, and zeta potential measurement have confirmed physicochemical properties of the prepared adsorbent. Furthermore, the data were evaluated using different equilibrium and kinetic models to comprehend the mechanism of phenol adsorption onto both adsorbents. Finally, lead ferrite-MAC regeneration experiments were carried out to assess the prepared adsorbent from the practical aspect.

## Experimental procedure

### Materials and reagents

1000 mg/L stock phenol solution was prepared using a specific amount of phenol (Merck, Darmstadt, Germany) in double-distilled water and mixed on a magnetic stirrer at 300 rpm. Then, it was diluted to prepare the desired concentration of phenol solution. Activated carbon (P60, Hanil, Korea) was used with no further purification. All other chemical reagents [HCl, NaOH, FeCl_3_, PbCl_2_, and oleic acid (Sigma–Aldrich)] were analytical grade.

### Lead ferrite-MAC preparation

0.4 mol/L (25 mL) of Fe^3+^ solution and 0.2 mol/L (25 mL) of Pb^2+^ solution were mixed in double-distilled water under magnetic stirring. 2.5 g of powdered activated carbon (PAC) was dispersed in 25 mL DD water under vigorous stirring. Then, the solution containing Fe^3+^ and Pb^2+^ was poured into 25 mL of the PAC solution^26^. After that, a solution of NaOH (25 mL) was added to the system with continuous magnetic stirring for 6 h until reaching the pH > 12. 3–4 drops (for 100 mL solution) of oleic acid were added with continuous magnetic stirring^36^. The obtained precipitate was then washed with DD water and ethanol to eliminate impurities. Finally, the obtained composite was separated and oven-dried at 60 °C. Finally, to complete the crystallization of the metals, the developed material was pyrolyzed in a muffle furnace at 700 °C for 1 h with a heating rate of 10 °C/min under an argon atmosphere. The formed material of lead ferrite-MAC was evaluated for phenol adsorption from the aqueous solutions.

### Lead ferrite-MAC characterization

The XRD pattern was determined using an X-ray diffractometer 1140 using Cu Kα radiation. The surface chemical characterizations of the prepared adsorbent were scanned from 400 to 4000 cm^−1^ using FTIR spectroscopy (Shimadzu IR instrument). The samples were analyzed morphologically by scanning electron micrographs (SEM, Seron Technology, AIS2100). The N_2_ adsorption–desorption isotherms (Belsorp mini II model) were applied to obtain the specific surface area and pore size of the developed adsorbent. Magnetic characterization of the developed adsorbent was carried out by the vibrating-sample magnetometer (VSM) at ambient temperature. In addition, zeta potential measurements at an initial phenol concentration of 100 mg/L were carried out to determine the point of zero charges (pH_pzc_) (Zetasizer Nano ZS, Malvern Instruments Inc., UK). The Smoluchowski equation (Eq. 1) calculated the zeta potential (ξ):

1 $$\mu =\frac{\xi \varepsilon V}{4\pi \eta d}$$

where $\mu$ is the electrophoretic mobility; $\varepsilon$ and d are the dielectric constant of medium and the electrode separation, respectively; $\eta$ and V are the viscosity of the suspension and the applied voltage, respectively^37, 38^.

### Batch adsorption experiment

All adsorption tests were carried out in a glass vessel sealed with a rubber cover at ambient temperature. The vessels were compressed with an aluminum cover to avoid the phenol losses by volatilization^39^. Few drops of dilute NaOH and HCl solutions were applied to regulate the solution acidity. After equilibrium, the mixture was filtered, and the phenol concentrations in the aqueous solutions were measured using spectrophotometry at 271 nm. The tests were repeated three times, and their mean values were considered as outputs. All other experimental conditions are depicted in the figure or table captions. The adsorbed quantity of phenol (*q*_*e*_) was calculated as follows (Eq. 2):

2 $${q}_{e}=\frac{({C}_{0}-{C}_{e})V}{m}$$

*C*_*0*_ and *C*_*e*_ represent the equilibrium and initial phenol concentrations in solutions (mg/L), *m* indicates the adsorbent quantity (g), and *V* denotes the aqueous phase volume (L).

Adsorption isotherm experiments for phenol adsorption were conducted in a beaker containing various initial nitrate concentrations with the adsorbent dosage of 1.5 g/L at ambient temperature and pH 7.0. In this research, the adsorption of phenol onto the adsorbents was evaluated by Dubinin–Radushkevich, Redlich-Peterson, Temkin, Freundlich, and Langmuir models. The coefficient of determination (R^2^)^40, 41^, mean square error (MSE)^42^, and average relative error (ARE)^43^ were applied as statistical criteria to evaluate the best fit to the experimental data.

Desorption experiments were carried out to evaluate the reusability of the developed adsorbent. In this context, 0.15 g of the adsorbent was added to 100 mL of phenol solution (500 mg/L) at ambient temperature (25 °C) and stirred for 4 h. Then, the adsorbent was separated from the solution and treated with 1 mol/L NaOH solution. Afterward, the used adsorbent was dried in an oven at 100 °C for 30 min. The adsorption–desorption cycles were repeated 6 times by using a similar adsorbent and initial phenol concentration.

## Results and discussion

### Lead ferrite-MAC features

**Figure 1:**
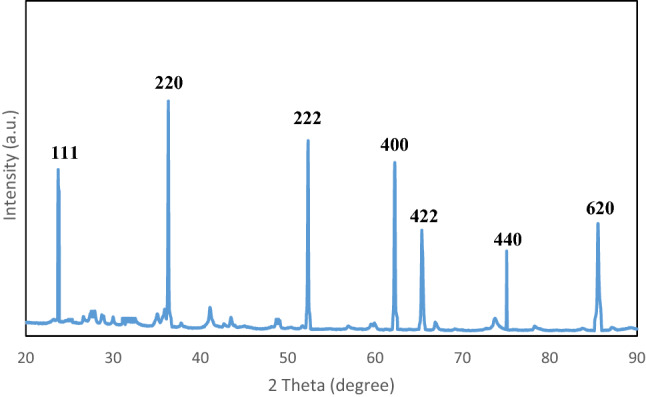
XRD pattern of lead ferrite-MAC.

The XRD pattern of the adsorbent (lead ferrite-MAC) is shown in Fig. 1. The prominent peaks representing 23.71°, 36.22°, 52.33°, 62.25°, 65.34°, 75.27°, and 85.51° were assigned to the planes of (111), (220), (222), (400), (422), (440), and (620), respectively. These peak positions are related to the presence of the spinel of the cubic structure. The spectra are in close agreement with JCPDS card No.04-0705 and with the findings of previous reports^44, 45^.

**Figure 2:**
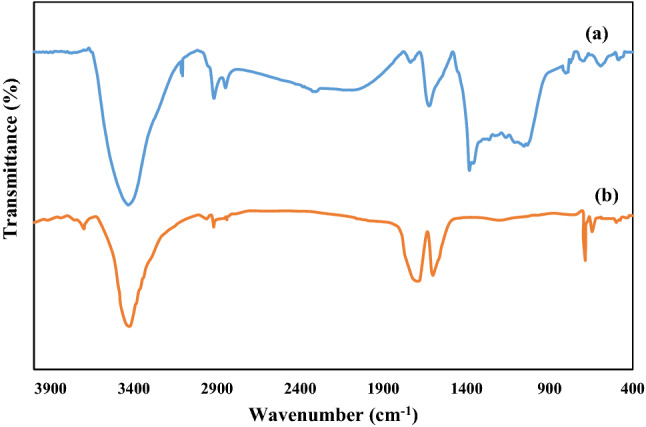
FTIR spectra of lead ferrite-MAC (**a**) after and (**b**) before phenol adsorption.

Infrared spectroscopy provides information on the functional groups at the adsorbent surface. In this context, FTIR spectra of adsorbent before and after phenol adsorption were performed (Fig. 2). Two bands at 1601.65 cm^−1^ and 3402.32 cm^−1^ were related to the bending vibration of H–O and absorbed water on the sample surface, respectively. The bands that appeared at about 1110–1180 cm^−1^ were related to C–O and C–O–C bonds’ vibration for activated carbon^46, 47^. The weak absorption band at 2920 cm^−1^ is shown, allotted to the symmetrical and asymmetrical stretching vibration of the CH_2_ group. In addition, the bands at 2840–2940 cm^−1^ were related to the C–H stretch. Two bands at 643 and 687 cm^−1^ are related to asymmetric bending vibration of Pb–O-Pb and Fe metal oxide (M–O) bond, respectively^48^. These findings confirmed the presence of Pb and Fe on the adsorbent surface.

Some changes were observed in the spectrum of lead ferrite-MAC adsorbent after phenol adsorption (Fig. 2b). The vibration mode for Pb–O–Pb and Fe–O–Fe shifted from 643 and 687 to 584 and 588 cm^−1^. The new band at 1384 cm^−1^ indicated the phenolic structure presence. Moreover, the band appearing at about 3110–2938 cm^−1^ was related to the phenolic type C–H. A mono-substituted aromatic structure appears at 807 and 774 cm^−1^ in the fingerprint region after phenol adsorption.

**Figure 3:**
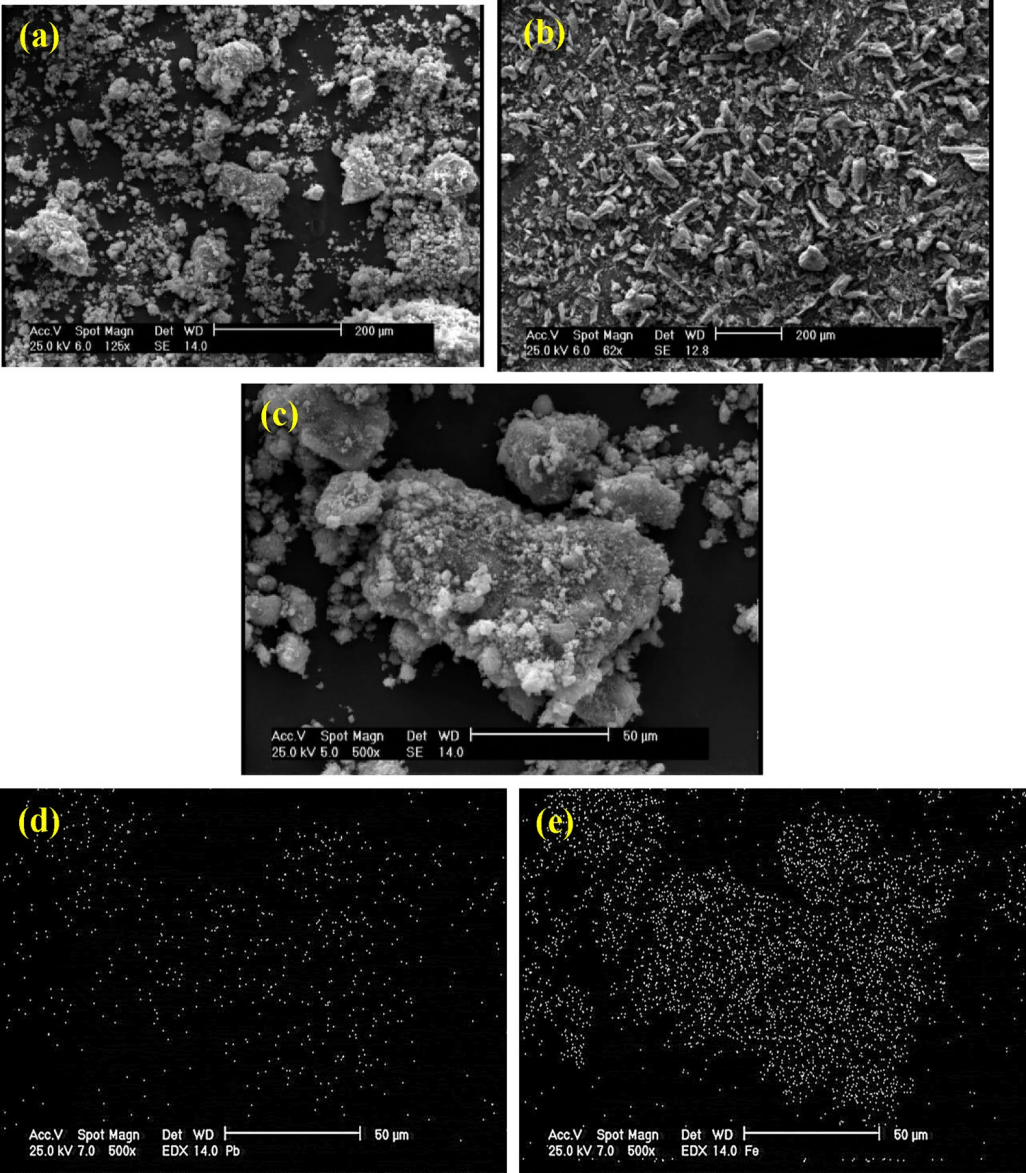
(**a**) and (**c**) SEM images of lead ferrite-MAC adsorbent at different scales, (**b**) SEM image of pristine activated carbon (**d**) X-ray mapping of Pb, (**e**) X-ray mapping of Fe in the lead ferrite-MAC composite.

To study the morphology of lead ferrite-MAC composite and pristine activated carbon, scanning electron microscopy analysis was applied. Figure 3a,c show the SEM photographs of the lead ferrite-MAC composite at different magnifications. Furthermore, by comparing Fig. 3a,b, it can be seen that the lead ferrite-MAC composite has more spherical particles than pristine activated carbon. In fact, pristine activated carbon has sharp-edged particles. It can be seen that the precipitates of lead and iron were aggregated together on the surface of AC. X-ray mapping in the SEM analyses can be used to identify the elemental distribution on the sample's surface. As shown in Fig. 3d,e, only elements Pb and Fe were found. It can be found that they were homogeneously distributed in the lead ferrite-MAC composite.

**Figure 4:**
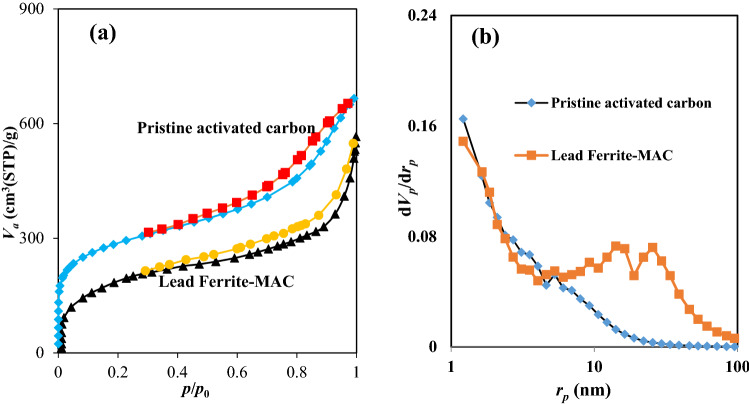
(**a**) N_2_ adsorption/desorption isotherm and (**b**) BJH pore size distributions of lead ferrite-MAC.

Figure 4 demonstrates the N_2_ adsorption and desorption isotherm for both adsorbents. Based on the IUPAC classification, both adsorbents have the pores of Type IV with an H3 hysteresis loop, representing that the adsorbents have a mesoporous structure with some microporous. The hysteresis loop at high relative pressure signifies the possible mesopores' nature. Furthermore, a small number of micropores within the developed adsorbent (lead ferrite-MAC) was seen at a pressure of around 0 to around 0.2. The pore size distribution plot of the adsorbent is shown in Fig. 4b. This curve indicates that the pores of the lead ferrite-MAC sample are generally distributed within multi-scales. For lead ferrite-MAC composite, two peaks at 14 and 25 nm and the peak ranging from 1 to 2 nm imply mesopore and micropore regions, respectively. Also, it can be found that pristine activated carbon and lead ferrite-MAC composite have a specific surface areas of 1023.9 and 774.53 m^2^/g and pore sizes of 4.013 and 11.89 nm, respectively. The total pore volume of lead ferrite-MAC adsorbent decreased a little, which may attribute to the blockage of these pores by metal ferrites after precipitation of lead and iron on the surface of activated carbon. However, the pore size of both adsorbents is very bigger than the phenol molecular size enabling its transference inside the adsorbent.

**Figure 5:**
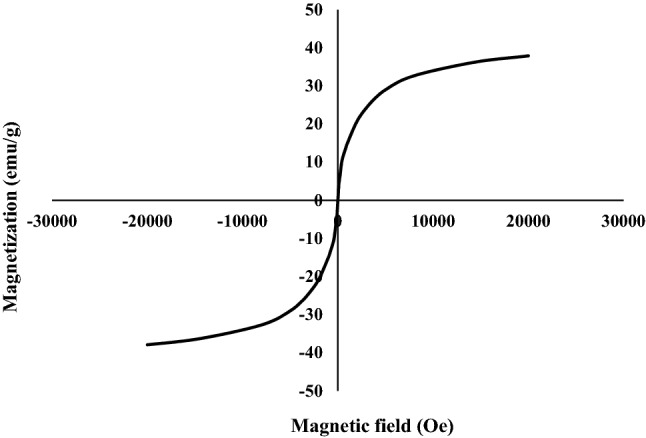
Hysteresis curve of lead ferrite-MAC.

The relative magnetization curve of lead ferrite-MAC was presented in Fig. 5. This curve was used to investigate the magnetic feature of the adsorbent. As a result, it was verified that the saturation magnetization of the developed adsorbent was found to be 37.9 emu/g. Furthermore, it was found that the permanent magnet could separate the developed adsorbent from the aqueous solutions. The saturation magnetization of lead ferrite-MAC composite is the range of saturation magnetization reported for other metal ferrites^27, 29^. The magnetic metal ferrites when compared with the magnetic iron oxide particles have several important advantages. These materials may have a narrow size distribution, whereas direct production of iron oxide nanoparticles leads to a widespread size range. In addition, they exhibit a higher specific loss power than the iron oxide nanoparticles, which makes them interesting as potential candidates for wastewater treatment purposes.

**Figure 6:**
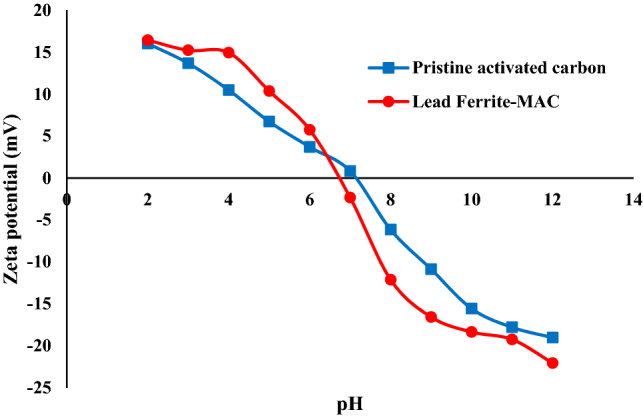
Zeta potential of lead ferrite-MAC and pristine AC versus pH value.

Figure 6 shows the zeta potential and pH_PZC_ on the surface of lead ferrite-MAC and pristine AC. The values of zero point charge for lead ferrite-MAC and pristine activated carbon were about 6.7 and 7.1, respectively. Thus, the surface of the adsorbent presents negative sites to adsorb cations when pH_PZC_ < pH, whereas in the case of pH < pH_PZC_ the positive sites are presented to adsorb anions^49, 50^.

Given the toxicity of Pb and Fe elements, it is necessary to evaluate the leaching of Pb and Fe into water. According to Environmental Protection Agency (EPA), the permissible levels of lead and iron in drinking water are 0.05 and 0.1 mg/L, respectively. In this regard, a series of experiments were carried out in a glass vessel sealed with a rubber cover at ambient temperature. The adsorbent and water (without phenol) were mixed with constant stirring using a magnetic stirrer at a fixed value of pH (2–14) for 24 h. Then, the concentrations of Pb and Fe were measured with an Agilant ICP-AES spectrometer. It can be found that at different values of pH, the concentration of Pb and Fe are well below the permissible limit based on EPA. So, this high stable adsorbent can be successfully used for wastewater treatment purposes.

### Effect of pH on the phenol adsorption

**Figure 7:**
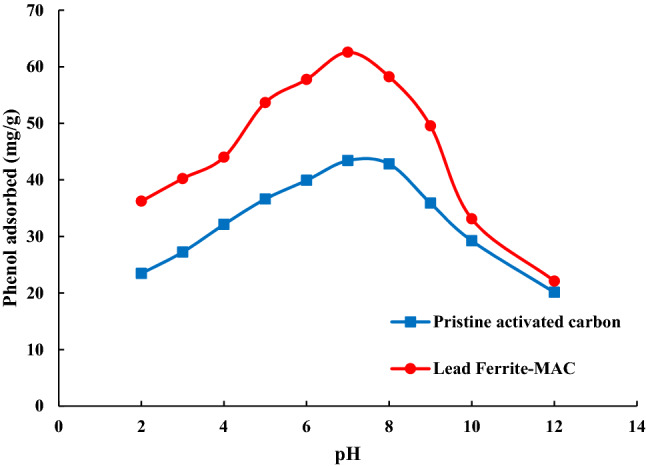
Influence of solution acidity on the phenol adsorption onto lead ferrite-MAC and pristine AC (25 °C, adsorbent dosage = 1.5 g/L).

Solution acidity is the most important variable influencing the process. Indeed, solution acidity affects the surface charge of the sorbent surface and the state of phenol that existed in the solution. The adsorption capacity of phenol onto lead ferrite-MAC and pristine AC was investigated at an initial pH range of 2.0–12.0 (Fig. 7). The extent of phenol adsorbed onto lead ferrite-MAC increased from 36.23 to 62.56 mg/g by increasing the pH of the solution from 2.0 to 7.0, respectively. In addition, the amount of adsorption capacity for pristine AC increased from 23.45 to 43.41 mg/g by increasing the pH of the solution from 2.0 to 7.0, respectively. When the solution pH decreases from about 7.0 to 2.0, the H^+^ concentration increases. The presence of more H^+^ in the solution makes the condition unfavorable for adsorption of phenol, as emerges a competition between H^+^ and phenol for occupying the adsorption sites. Albeit, further increase in pH, beyond the value of 7.0, decreased the adsorption capacity of both adsorbents. As mentioned, the zero-point charge values of lead ferrite-MAC and pristine AC were about 6.7 and 7.1, respectively, above which the adsorbent surface presents negative charges. Besides, at the higher values of pH, the presence of higher OH^−^ in the solution cause the dissociation of H^+^ from the phenol, leaving them in the anionic dominant form. Therefore, a repulsive electrostatic interaction prevailed that resulted in a reduction in the capacity of adsorption. Thus, there would be an optimum pH condition (about 7 for the current study) around which the maximum uptake is usually expected.

### Equilibrium studies

**Figure 8:**
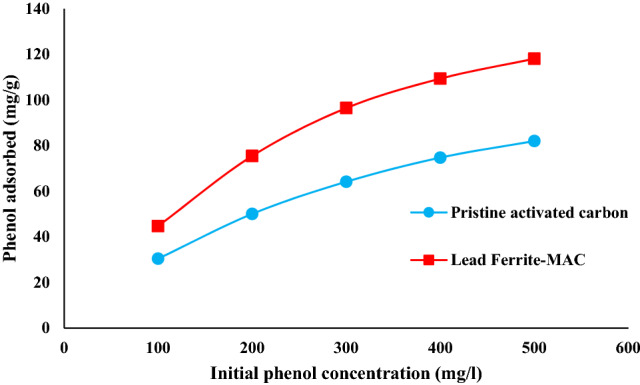
Influence of concentration on the phenol adsorbed onto lead ferrite-MAC and pristine AC (pH 7, adsorbent dosage = 1.5 g/L, 25 °C).

The relationship between the quantity of phenol adsorbed onto lead ferrite-MAC and pristine AC, and the phenol concentration in the equilibrium state at a fixed temperature presents an adsorption isotherm. Adsorption isotherm provides essential information about the capability of the adsorption process for wastewater decontamination applications^13^. According to Fig. 8, the extent of phenol absorbed onto both adsorbents enhances by increasing the concentration of phenol until it reaches a plateau area, representing the maximum adsorption capacity of lead ferrite-MAC and pristine AC. This shape is a favorable type of isotherm and indicates that lead ferrite-MAC and pristine AC are good adsorbents for phenol.

**Figure 9:**
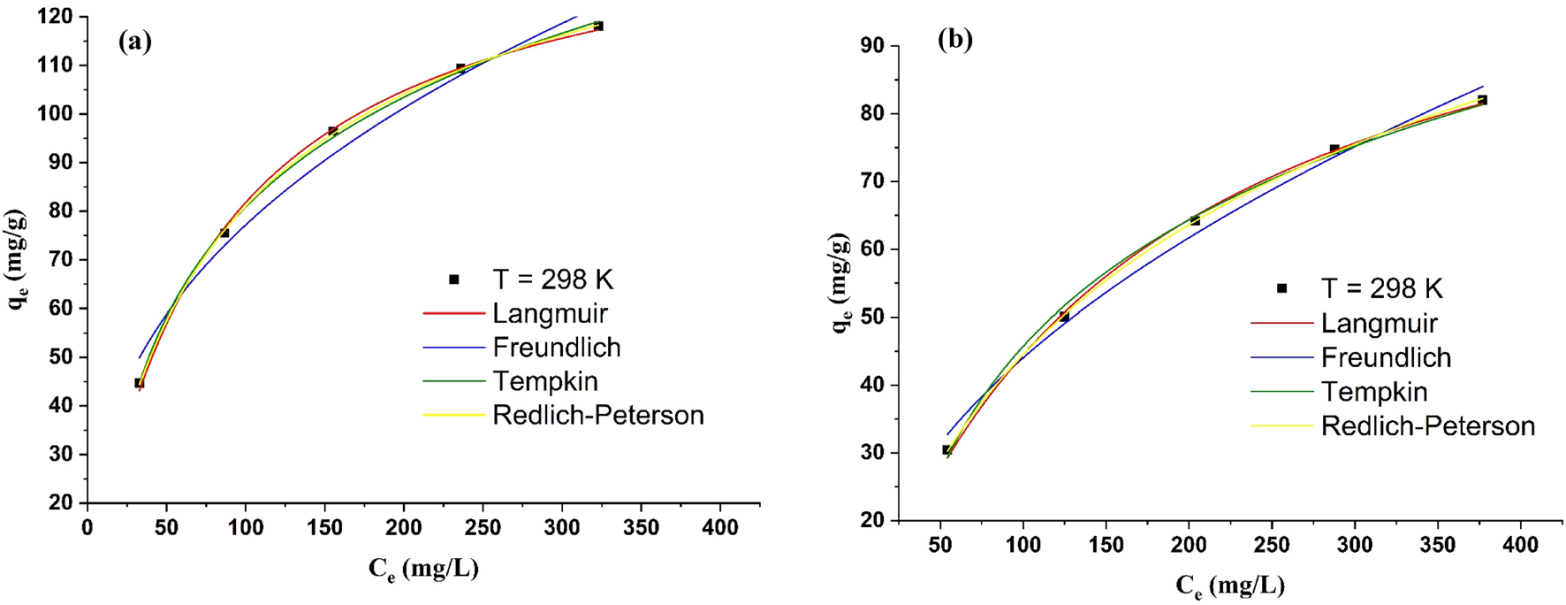
Isotherm curve of phenol adsorption by (**a**) lead ferrite-MAC and (**b**) pristine AC (pH 7, adsorbent dosage = 1.5 g/L, 25 °C).

There are different isotherm models in the literature to represent the equilibrium curves and extract information about the system^13, 51^. In this research, Freundlich, Langmuir, Redlich-Peterson, and Tempkin isotherms were applied to explain the equilibrium adsorption (Fig. 9). The Langmuir isotherm assumes that all adsorption sites have the same sorption activation energy and have no transmigration in the plane of the surface^52^. The Freundlich isotherm was derived from the assumption that adsorption occurs on a heterogeneous surface through a multilayer adsorption mechanism^53^. The Tempkin isotherm considers the adsorbate–adsorbent interactions^54^. These interactions decrease the heat of molecular adsorption in the layer. The distribution of binding energies determines the adsorption process^54^. Redlich-Peterson^55^ behaves like the Freundlich isotherm at high concentrations of sorbate, and at a low concentration, it approximates Henry’s law. Their nonlinear equations are presented as follows:

3 $$q_{e} = \frac{{q_{m} K_{L} C_{e} }}{{1 + K_{L} C_{e} }}$$

4 $$q_{e} = K_{F} C_{e}^{1/n}$$

5 $$q_{e} = B\ln (K_{T} C_{e} )$$

6 $$q_{e} = \frac{{K_{RP} C_{e} }}{{1 + a_{RP} C_{e}^{\beta } }}$$

where *q*_*m*_ is the theoretical saturation capacity (mg/g); *K*_*L*_ is the Langmuir constant (L/mg); *K*_*F*_ is the constant of the Freundlich model indicating the adsorbent’s adsorption capacity (mg/g)(mg/L)^−1/n^; *1/n* is the Freundlich exponent; *B* (defined as *RT/b*_*T*_) (mg/g) is the Tempkin constant corresponding to the adsorption heat; *K*_*T*_ is a constant related to maximum binding energy (L/mg); *T* is temperature; *R* denotes the universal gas constant; *K*_*RP*_ (L/g), *a*_*RP*_ (L/mg)^β^), and *β* are constants in Redlich-Peterson isotherm.

Table 2 shows the equilibrium parameters for the systems of phenol/lead ferrite-MAC and phenol/pristine AC, according to Eqs. (3–6).

**Table 2:** Isotherm parameters for phenol adsorption by lead ferrite-MAC and pristine AC.

Model	Parameter	
	Lead ferrite-MAC	Pristine AC
**Langmuir**		
*q*_*m*_ (mg/g)	145.708	116.606
*K*_*L*_ (L/mg)	0.013	0.006
*R*^2^	0.9986	0.9984
*R*^2^_*adj*_	0.9982	0.9979
*ARE* (%)	1.872	1.429
*MSE* (mg/g)^2^	0.941	0.529
**Freundlich**		
*K*_*F*_ ((mg/g)(mg/L)^−1/nF^)	12.713	4.688
*1/n* (dimensionless)	0.392	0.486
*R*^2^	0.97863	0.9811
*R*^2^_*adj*_	0.97151	0.9782
*ARE* (%)	7.228	5.269
*MSE* (mg/g)^2^	14.778	6.995
**Redlich-Peterson**		
*K*_*RP*_ (L/g)	2.155	0.882
*a*_*RP*_ (L/mg)^n^_RP_	0.024	0.019
*n*_*RP*_ (dimensionless)	0.920	0.852
*R*^2^	0.9997	0.9998
*R*^2^_*adj*_	0.9995	0.9995
*ARE* (%)	0.724	0.423
*MSE* (mg/g)^2^	0.178	0.078
**Tempkin**		
*K*_*T*_ (L/mg)	76.173	92.349
*b*_*T*_ (J/mol)	0.120	0.055
*R*^2^	0.9990	0.9969
*R*^2^_*adj*_	0.9987	0.9959
*ARE* (%)	1.086	1.893
*MSE* (mg/g)^2^	0.672	1.019

The parameter estimations were performed using proper script programming on Matlab 2017. Regarding the higher values of coefficient of determination (*R*^2^) and adjusted determination coefficient (*R*^2^_*adj*_), coupled with the lower values of average relative error (*ARE*) and minimum squared error (*MSE*) (Table 2), it can be found that the isothermal data for Pristine AC were in agreement with the isotherm models of Tempkin and Redlich-Peterson. As the number of surface groups and the phenol uptake are high for activated carbon, adsorbate–adsorbate interactions are of major importance, justifying the relevant application of the Tempkin equation. In addition, the isothermal data for lead ferrite-MAC were in agreement with the isotherm models of Redlich-Peterson and Langmuir. These results indicate that the phenol molecules are adsorbed by specific sites of the lead ferrite-MAC adsorbent, undergoing adsorption in a monolayer form. It has been found that the presence of metal hydroxides on the surface of AC increases the adsorption capacity of phenol on lead ferrite-MAC (Table 2). This can be explained in terms of the molecular coordination between phenol and metal hydroxides^18^ along with the active sites of AC. The Freundlich constants and 1/n values also confirmed that the present adsorption results for phenol on both adsorbents stemmed from physical and chemical interactions between the adsorbent and adsorbate.

According to isotherm parameters, the maximum adsorption capacity of the lead ferrite-MAC composite was higher than that of pristine AC, which was in agreement with adsorption experimental trends. This difference is due to the difference in the structure of the adsorbent because the composite of lead ferrite-MAC contains additional metal hydroxides.

The Dubinin–Radushkevich (D–R) model also performed the analysis of the equilibrium data. This analysis was realized to determine whether the adsorption process is chemical or physical^56^. This model assumes that ionic species binding follows by multilayer adsorption. D–R equation is as follows:

7 $$\mathrm{ln}({q}_{e})=\mathrm{ln}({q}_{s})-{K}_{DR}{\varepsilon }^{2}$$

8 $$\varepsilon =RT\mathrm{ln}\left(1+\frac{1}{{C}_{e}}\right)$$

Being *q*_*s*_ is the isotherm saturation capacity, and *K*_*DR*_ is the D–R constant. The mean free energy is defined as follows:

9 $$E=\frac{1}{\sqrt{-2{K}_{DR}}}$$

The plot of [*lnq*_*e*_] vs. [*ln*^2^*(1* + *1/C*_*e*_*)*] for the phenol adsorption onto lead ferrite-MAC and pristine AC presented coefficients of determination of 0.9917 and 0.9979, respectively. The *E* value for both adsorbents was relatively 4 kJ/mol. According to the D–R model, chemical adsorption is favored at mean free energy (*E*) between 8 and 16 kJ/mol, while for *E* < 8 kJ/mol, adsorption proceeds physically. Therefore, the adsorption of phenol onto both adsorbents may be conducted by a physical mechanism.

### Adsorption kinetics

**Figure 10:**
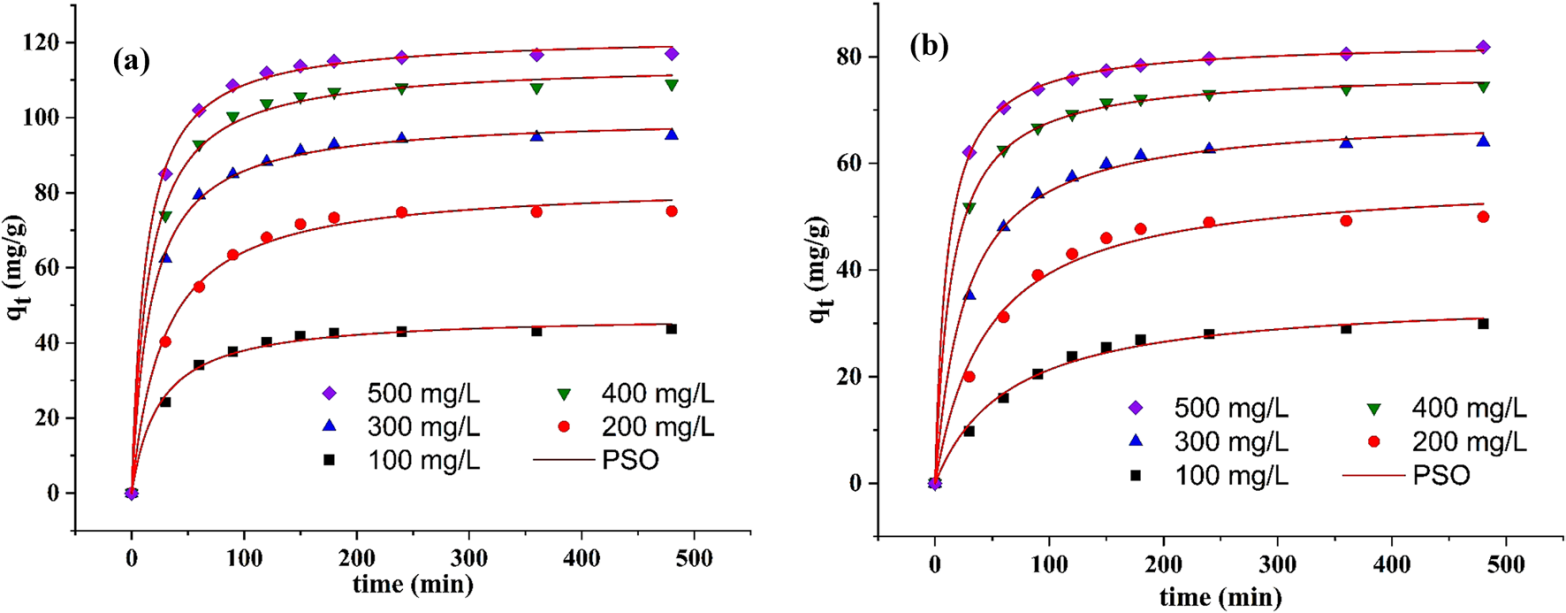
Kinetic curves of phenol adsorption by (**a**) lead ferrite-MAC and (**b**) pristine AC (pH 7, adsorbent dosage = 1.5 g/L, 25 °C).

To identify the necessary time for equilibrium, the phenol uptake onto lead ferrite-MAC and pristine AC at different initial phenol concentrations was examined versus contact time. The amount of phenol absorbed on both adsorbents was measured at 0, 30, 60, 90, 120, 150, 180, 240, 360, and 480 min. Figure 10 shows the phenol adsorption profiles onto lead ferrite-MAC and pristine AC at various initial phenol concentrations. The adsorption of phenol at the initial time increased drastically and reached approximately a constant value after 200 min. Therefore, the contact time of 180 min is enough to attain equilibrium. However, a contact time of 300 min was selected to ensure completed equilibrium.

The reaction models of pseudo-first-order (PFO) (Eq. 10), pseudo-second-order (PSO) (Eq. 11), and Elovich (Eq. 12)^55, 57^ were applied to comprehend better the adsorption mechanism according to the kinetic curves. All these models consider adsorption as a reaction. It is noted that this fact is not true, but sometimes, these models can complement the information about the adsorption process.

10 $${\text{q}}_{\text{t}}={\text{q}}_{1}{(1}-{\exp(}-{\text{k}}_{1}{\text{t}}{)}{)}$$

11 $${\text{q}}_{\text{t}}= \, \frac{\text{t}}{\left(\frac{1}{{\text{k}}_{2}{{\text{q}}}_{2}^{2}}\right){+}\left(\frac{\text{t}}{{\text{q}}_{2}}\right)}$$

12 $${\text{q}}_{\text{t}}{ = }\frac{1}{{\text{a}}}{\text{ln}}\left({1} + {\text{abt}}\right)$$

where *k*_1_ (1/min) and *k*_2_ (g/mg min) are the kinetics constants of PFO and PSO, respectively; *q*_1_ and *q*_2_ are the theoretical values for the adsorption capacity (mg/g); *b* is the initial velocity (mg/g min); *a* is the desorption constant of Elovich model (g/mg).

In addition to the reaction models, the linear driving force model (LDF)^58^, which is based on the mass transfer phenomena, was applied in this research. LDF model is given by:

13 $$\frac{{\text{d}}\stackrel{\mathrm{-}}{\text{q}}}{\text{dt}}={\text{k}}_{\text{LDF}}\left({\text{q}}^{*}-\stackrel{\mathrm{-}}{\text{q}}\right)$$

14 $$\stackrel-{\text{q}} \, \left(\text{t=0}\right)= \text{0}$$

where $\stackrel-{\text{q}}$ is the average phenol adsorption capacity (mg/g), ${\text{q}}^{*}$ is the phenol adsorption capacity related to the concentration in the liquid phase at equilibrium (mg/g^−1^), and *k*_*LDF*_ is the LDF mass transfer coefficient (1/min). In the case of this work, the Langmuir model presented the best fit for the equilibrium, so q* in Eq. (14) was substituted by Eq. (3). In addition, the global mass balance of phenol in the batch (Eq. 2) was inserted in Eq. (14), leading to:

15 $$\frac{{\text{d}}\stackrel-{\text{q}}}{\text{dt}}={\text{k}}_{\text{LDF}}\left({\text{q}}_{\text{m}}\frac{{\text{K}}_{\text{L}}\left({\text{C}}_{0}\text{-(m/V}\stackrel{\mathrm{-}}{\text{)q}}\right)}{\text{1} + {\text{K}}_{\text{L}}\left({\text{C}}_{0}\text{-(m/V)}\stackrel{\mathrm{-}}{\text{q}}\right)}-\stackrel{\mathrm{-}}{\text{q}}\right)$$

The parameter *k*_*LDF*_ can be related to the diffusivity inside the adsorbent (*D*_*h*_) and the particle radius (*R*) by^58^:

16 $${\text{D}}_{\text{h}}=\frac{{\text{R}}^{2}{{\text{k}}}_{\text{LDF}}}{15}$$

The kinetic parameters of the systems phenol/lead ferrite-MAC and phenol/pristine AC, estimated by the fitting of Eqs. (10), (11), (12), and (15) to the experimental data (Fig. 10), are depicted in Table 3. Among the reaction models, the PSO model was the best to represent the data, with higher values of coefficient of determination (*R*^*2*^) and adjusted determination coefficient (*R*^*2*^_*adj*_), and lower values of average relative error (*ARE*) and minimum squared error (*MSE*) (Tables 3, 4). The parameter *q*_*2*_ of such model for lead ferrite-MAC and pristine AC increased from 47.397 and 35.177 mg/g to 121.963 and 82.395 mg/g, respectively when the initial phenol concentration increased from 100 to 500 mg/L. However, the rate of this increase was successively lower. Both facts are related to the progressive increase in the surface coverage.

**Table 3:** Kinetic parameters of phenol adsorption by lead ferrite-MAC.

Model	Initial phenol concentration (mg/L)				
	100	200	300	400	500
**PFO**					
*q*_*1*_ (mg/g)	41.886	71.205	89.667	102.731	111.503
*k*_*1*_ (1/min)	0.0462	0.0433	0.0642	0.0769	0.0821
*R*^*2*^	0.9678	0.9465	0.9739	0.9771	0.9657
*R*^*2*^_*adj*_	0.9675	0.9460	0.9732	0.9768	0.9652
*ARE* (%)	5.426	6.986	4.455	6.697	4.020
*MSE* (mg/g)^2^	7.412	9.044	5.169	10.252	8.589
**PSO**					
*q*_*2*_ (mg/g)	47.397	82.694	100.389	114.702	121.963
*k*_*2*_ (g/mg min)	8.531 × 10^–4^	4.203 × 10^–4^	5.860 × 10^–4^	5.844 × 10^–4^	6.706 × 10^–4^
*R*^*2*^	0.9941	0.9944	0.9986	0.9974	0.9989
*R*^*2*^_*adj*_	0.9933	0.9937	0.9985	0.9970	0.9988
*ARE* (%)	2.600	2.556	1.170	1.598	0.975
*MSE* (mg/g)^2^	1.112	3.153	1.179	3.000	1.340
**Elovich**					
*a* (g/mg)	0.151	0.080	0.089	0.086	0.093
*b* (mg/g min)	16.860	17.449	186.604	499.364	1988.181
*R*^*2*^	0.9672	0.9684	0.9827	0.9797	0.9866
*R*^*2*^_*adj*_	0.9631	0.9644	0.9805	0.9772	0.9849
*ARE* (%)	6.178	6.151	4.206	4.433	3.497
*MSE* (mg/g)^2^	6.145	17.805	14.860	22.968	17.327
**LDF**					
*k*_*LDF*_ (1/s)	2.93 × 10^–4^	4.46 × 10^–4^	7.37 × 10^–4^	8.17 × 10^–4^	9.02 × 10^–4^
*D*_*h*_ (cm^2^/s)	4.41 × 10^–9^	8.96 × 10^–9^	1.83 × 10^–8^	1.94 × 10^–8^	2.06 × 10^–8^
*R*^*2*^	0.9666	0.9588	0.9748	0.9841	0.9906
*R*^*2*^_*adj*_	0.9624	0.9535	0.9716	0.9821	0.9894
*ARE* (%)	5.43	6.06	5.01	4.02	3.05
*MSE* (mg/g)^2^	5.99	9.28	10.54	9.23	7.15

**Table 4:** Kinetic parameters of phenol adsorption by pristine AC.

Model	Initial phenol concentration (mg/L)				
	100	200	300	400	500
**PFO**					
*q*_1_ (mg/g)	16.229	22.228	60.125	67.749	73.952
*k*_1_ (1/min)	0.012	0.016	0.028	0.025	0.024
*R*^2^	0.9013	0.9001	0.9913	0.9777	0.9578
*R*^2^_*adj*_	0.9008	0.8989	0.9902	0.9768	0.9752
*ARE* (%)	8.738	9.616	3.902	5.494	4.900
*MSE* (mg/g)^2^	14.412	15.986	1.977	9.252	7.589
**PSO**					
*q*_2_ (mg/g)	35.177	57.401	69.321	77.442	82.935
*k*_2_ (g/mg min)	4.365 × 10^–4^	3.782 × 10^–4^	5.396 × 10^–4^	8.920 × 10^–4^	1.160 × 10^–3^
*R*^2^	0.9912	0.9877	0.9974	0.9997	0.9998
*R*^2^_*adj*_	0.9901	0.9862	0.9971	0.9996	0.9998
*ARE* (%)	4.316	4.641	1.647	0.490	0.391
*MSE* (mg/g)^2^	0.831	3.243	1.017	0.169	0.119
**Elovich**					
*a* (g/mg)	0.151	0.080	0.089	0.086	0.093
*b* (mg/g min)	16.860	17.449	186.604	499.364	1988.181
*R*^2^	0.9672	0.9684	0.9827	0.9797	0.9866
*R*^2^_*adj*_	0.9631	0.9644	0.9805	0.9772	0.9849
*ARE* (%)	11.863	10.384	6.749	6.490	4.992
*MSE* (mg/g)^2^	6.145	17.805	14.860	22.968	17.327
**LDF**					
*k*_*LDF*_ (1/s)	1.97 × 10^–4^	2.99 × 10^–4^	3.81 × 10^–4^	4.02 × 10^–4^	4.62 × 10^–4^
*D*_*h*_ (cm^2^/s)	1.41 × 10^–9^	8.05 × 10^–9^	2.25 × 10^–8^	4.41 × 10^–8^	8.43 × 10^–8^
*R*^2^	0.9723	0.9634	0.9571	0.9769	0.9682
*R*^2^_*adj*_	0.9701	0.9612	0.9489	0.9747	0.9667
*ARE* (%)	4.213	6.108	10.183	5.550	5.812
*MSE* (mg/g)^2^	3.871	4.104	6.586	5.707	5.234

In the same way, the parameter *k*_*2*_ for lead ferrite-MAC and pristine AC raised from 8.531 × 10^–4^ and 4.365 × 10^–4^ g/mg min to 6.706 × 10^–4^ and 1.160 × 10^–3^ g/mg min, respectively with the increase in initial phenol concentration (Tables 3, 4). This behavior shows that the adsorption phenomenon was progressively faster when the initial concentration increased. This fact is explained by the external and internal concentration gradients that are higher at higher phenol concentrations.

The LDF mass transfer model could also explain the phenol adsorption on both adsorbents. In this sense, it can be found that the phenol adsorption on lead ferrite-MAC and pristine activated carbon occurs according to the diffusion in a homogeneous adsorbent from the mass transfer aspect. This mechanism was also assumed by Franco et al.^59^, studying the phenol adsorption on activated carbon prepared from fruit wastes of the *Ceiba speciosa*. The values of *k*_*LDF*_ and *D*_*h*_ corroborated the same trend of *k*_*2*_. Besides, *D*_*h*_ for lead ferrite-MAC and pristine activated carbon ranged from 4.41 × 10^–9^ and 1.41 × 10^–9^ to 2.06 × 10^–8^ and 8.43 × 10^–8^ cm^2^/s, respectively. These values are in the same range as those found by Franco et al.^59^ (activated carbon) and Ocampo-Pérez et al.^60^ (bituminous coal).

### Proposed mechanism of phenol adsorption

According to isotherm parameters, the maximum adsorption capacity of lead ferrite-MAC composite was more than that of pristine activated carbon, which was in agreement with adsorption experimental trends. This difference is due to the metal hydroxides coated on activated carbon since they improve the retention of phenol on the available active sites of the adsorbent and create an additional electrostatic interaction with the phenol adsorbate. Electrostatic attraction between metal hydroxides and the acidic –OH of phenol can be one of the possible mechanisms of phenol adsorption onto lead ferrite-MAC composite^61^. Some changes in the bending vibration of M–O before and after phenol adsorption (FTIR analysis) confirmed the electrostatic interaction of phenol with lead ferrite-MAC. Another possible mechanism of phenol adsorption onto the developed adsorbent is hydrogen-bonding interactions between the functional groups of activated carbon (CO and OH) with phenolic –OH. In addition, π–π electron donor–acceptor interactions between aromatic rings of phenol and the surface of the adsorbent affect the adsorption process^62^.

### Comparison with other adsorbents and reusability of lead ferrite-MAC

Selective phenol adsorption using different adsorbents like activated carbon^22^, biological materials^31^, and minerals^14^ has been investigated. Table 5 indicates the comparison of phenol removal using different adsorbents. The adsorption capacity of the lead ferrite-MAC adsorbent in this research is not maximum. However, it is better than some adsorbents such as magnetic activated carbon cobalt nanoparticles; tithonia diversifolia activated carbon, biochar produced from an oil palm frond, diethylenetriamine-modified activated carbon, activated carbon obtained from black wattle bark waste, NiFe_2_O_4_-powdered activated carbon, and biochar made of the pine fruit shells (BC550). Therefore, it can be concluded that the adsorbent prepared here is promising and competitive.

**Table 5:** Comparison of phenol adsorption using diverse adsorbents.

Adsorbent	q_m_ (mg/g)	References
Activated carbon from fruit wastes of the *Ceiba speciosa*	156.7	^59^
Activated carbon/zirconium oxide composite	166.7	^67^
Magnetic activated carbon cobalt nanoparticles	107.50	^64^
Tithonia diversifolia activated carbon	50.55	^68^
Biochar produced from an oil palm frond	62.89	^69^
Diethylenetriamine-modified activated carbon	18.12	^70^
Activated carbon obtained from black wattle bark waste	98.57	^71^
NiFe_2_O_4_-powdered activated carbon	93.25	^72^
Biochar obtained from the pine fruit shells (BC550)	26.74	^73^
Pristine activated carbon	116.61	Present study
Lead ferrite-magnetic activated carbon	145.71	Present study

**Figure 11:**
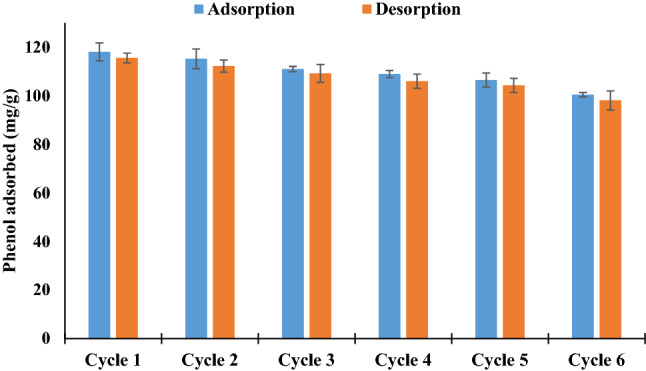
Phenol adsorbed onto lead ferrite-MAC in successive cycles (*C*_0_ = 500 mg/L, 4 h, 25 °C, pH 7, adsorbent dosage = 1.5 g/L).

Another attribute of lead ferrite-MAC that makes it a promising material is reusability. The reusability of an adsorbent is a crucial factor from the practical aspect, making it a commercially attractive and green method. Different methods include heating regeneration, solvent washing, and chemical and petrochemical regeneration for regenerating adsorbents^63, 64^. Among them, solvent washing is a well-known method for recovering and reusing the adsorbent. So, the solvent washing method was applied to regenerate the developed adsorbent. The regeneration experiments of lead ferrite-MAC were conducted for six cycles using 1 mol/L NaOH. When washing with NaOH, sodium phenolate is formed because of the reaction of sodium hydroxide with phenol. This product can be easily desorbed and dissolved in the solution of solvent^65, 66^. The obtained results are presented in Fig. 11. The adsorption capacity of phenol onto the adsorbent decreased with the number of cycles. This phenomenon have resulted from the saturation of active sites on the surface of the adsorbent, the protonation of available functional groups on the surface of the adsorbent, and/or the destruction of adsorbent particles during successive cycles of adsorption/desorption. However, the adsorbent maintained 85% of this maximum potential after these cycles. It can be found that lead ferrite-MAC has interesting adsorption potential after six cycles of regeneration. The results confirmed the practical usefulness of the lead ferrite-MAC composite.

## Phenol removal from the real industrial wastewater

To evaluate the practical application of the lead ferrite-MAC adsorbent for the removal of phenol from the real industrial wastewater, 0.15 g of the lead ferrite-MAC composite and pristine activated carbon was stirred with 100 ml of wastewater sample taken from the nearby industrial area of Parsian, Bandarabbas, Iran. To remove solid particles and impurities, the wastewater sample was filtered. The wastewater sample was analyzed for the contents of pollutants. The major pollutants of this sample were phenol, copper, chromium, nickel, zinc, cobalt, manganese, and arsenic as presented in Table 6. It is noted that the original sample spiked with 90 μg/L of phenol. The experiment was carried out at an ambient temperature, stirring speed of 300 rpm, and a stirring time of 400 min.

**Table 6:** Competitive phenol adsorption from the real industrial wastewater using lead ferrite-MAC and pristine AC.

Parameters	C_i_ (μg/L)	C_f_ (μg/L)		Removal efficiency (%)	
		AC	Lead ferrite-MAC	AC	Lead ferrite-MAC
Phenol	98.83	19.32	4.12	80.45	95.83
Copper	23.76	6.12	3.23	74.24	86.41
Chromium	9.7	2.11	1.12	78.25	88.45
Nickel	12.67	4.03	1.27	68.19	89.98
Zinc	7.83	2.01	0.93	74.33	88.12
Cobalt	10.04	2.12	1.22	78.88	87.85
Manganese	7.32	1.98	Not detected	72.95	100.00
Arsenic	5.1	0.92	Not detected	81.96	100.00

The developed adsorbent (lead ferrite-MAC) showed significant adsorption efficiency for several pollutants. Results indicated that the lead ferrite-MAC composite could efficiently remove phenol from waste streams and the removal was relatively 96%. Furthermore, the developed adsorbent removed other pollutants like copper, chromium, nickel, zinc, cobalt, manganese, and arsenic with efficiencies of relatively 90%. Therefore, the developed adsorbent can be successfully applied with high efficiency for the practical removal of phenol from waste streams to produce healthy water using a stable and efficient adsorbent.

## Conclusions

In this research, a novel lead ferrite-activated carbon composite (lead ferrite-MAC) was developed and compared with pristine AC for the uptake of phenol, where the most influential operating parameters were analyzed. Instrumental analyses such as XRD, SEM, FTIR, zeta potential, and BET analysis were carried out to characterize lead ferrite-MAC adsorbent. lead ferrite-MAC was preponderantly a mesoporous adsorbent, with some microporous, and presented a surface area of 747.53 m^2^/g and pore size of 11.89 nm. The zeta potential was 6.7, and a saturation magnetization of 37.9 emu/g proved the magnetic character. Concerning the phenol adsorption potential for both adsorbents, the best pH was 7.0. Results indicated that the maximum adsorption capacities for lead ferrite-MAC composite and pristine activated carbon were 145.708 and 116.606 mg/g, respectively. Regarding the high value of the coefficient of determination (*R*^2^) and adjusted determination coefficient (*R*^*2*^_*adj*_), coupled with the lower values of average relative error (*ARE*) and minimum squared error (*MSE*), it can be found that the isothermal data for lead ferrite-MAC adsorbent were in agreement with the isotherm models of Redlich-Peterson and Langmuir. According to isotherm parameters, the maximum adsorption capacity of lead ferrite-MAC composite was more than that of pristine activated carbon, which was in agreement with adsorption experimental trends. This difference is due to the metal hydroxides coated on activated carbon since they improve the retention of phenol on the available active sites of the adsorbent and create an additional electrostatic interaction with the phenol adsorbate. The mean free energy for the systems of phenol/lead ferrite-MAC and phenol/pristine AC was relatively 4 kJ/mol, indicating a physical mechanism. Among the kinetic models, the PSO model was the best to represent the data, with higher values of coefficient of determination (R^2^) and adjusted determination coefficient (R^2^_adj_), and lower values of average relative error (ARE) and minimum squared error (MSE). The phenol adsorption onto lead ferrite-MAC and pristine AC was relatively fast and explained by the diffusion in a homogeneous solid. The diffusion coefficient values, *D*_*h*_, for lead ferrite-MAC and pristine AC ranged from 4.41 × 10^–9^ and 1.41 × 10^–9^ to 2.06 × 10^–8^ and 8.43 × 10^–8^ cm^2^/s, respectively. In addition, the adsorption capacity of lead ferrite-MAC is within the literature range, but the material can be reused 6 times. The results presented in this work point out that lead ferrite-MAC is a promising and competitive adsorbent to uptake phenol from aqueous media.

## Data Availability

The datasets used and/or analyzed during the current study available from the corresponding author on reasonable request.
